# Laparoscopic salvage total pelvic exenteration: Is it possible post-chemo-radiotherapy?

**DOI:** 10.4103/0972-9941.59310

**Published:** 2009

**Authors:** H Patel, J V Joseph, A Amodeo, K Kothari

**Affiliations:** Section of Laparoscopic Urology, Institute of Urology, University College Hospital, London, UK; 1 Section of Laparoscopy and Robotic Surgery, University of Rochester Medical Center, Rochester, NY, USA; 2 Section of Minimally Invasive Surgery, Gujarat Cancer and Research Institute, Ahmadabad, India

**Keywords:** Laparoscopy, pelvic exenteration, malignancy

## Abstract

Indications for total pelvic exenteration in a male (removal of the bladder, prostate and rectum) and in a woman (removal bladder, uterus, vagina, ovaries and rectum) are rare. The advanced stage generally dictates that the patient has some form of chemotherapy or radiotherapy, or a combination of two to shrink/debulk the tumour. We report the first two cases of a salvage laparoscopic total pelvic exenteration in a male for rectal adenocarcinoma invading into the bladder and prostate, post-chemo-radiotherapy and in a woman for squamous cell carcinoma of cervix invading the bladder and rectum post-chemo-radiotherapy. Salvage surgery is often difficult and has been noted to have high morbidity. Applying a laparoscopic approach to this group may have advantages for the patient and the surgeon, i.e. less pain, early recovery and magnified views. As we have technically shown it to be possible, perhaps laparoscopic approaches should be discussed if the teams in these centres are of advanced laparoscopic surgeons working in multi-skilled groups.

## INTRODUCTION

Laparoscopic surgery has advanced considerably in recent years. The indications for its use have widened and the superseding of open surgery seems inevitable in many areas of surgery. This revolution in surgery is in part associated with the incredible technological advancement and also the advanced skills acquired by surgeons gifted in the field of laparoscopy. The multi-skilled teams working in major laparoscopic surgical centres have made these advances constantly.

The advances in robotic surgical technologies are also occurring. However, these technologies are currently being used as enhancement devices, and do not replace the intuitive surgical skills acquired by the advanced pure laparoscopist.

The indications for a total pelvic exenteration are rare. The advanced stage generally dictates that the patient has some form of chemotherapy or radiotherapy, or a combination of two to shrink/debulk the tumour. The surgical option before or after chemo-radiotherapy is a challenging issue for open surgeons. Thus, to contemplate a laparoscopic approach for this condition requires good planning and discussion, as well as the appropriate advanced skill set. However, as with most limitations to a new technique, in time and with skill acquisitions it may be possible.

We report the first two cases of a salvage laparoscopic total pelvic exenteration in a male for rectal adenocarcinoma invading into the bladder and the prostate, post-chemo-radiotherapy and in a woman for squamous cell carcinoma of cervix invading into the bladder and the rectum post-chemo-radiotherapy.

## CASE REPORTS

### Case 1

A male aged 40 years presented with painful defecation over a period of 2 months. He did not complain of any other systemic or organ-specific problem such as lower urinary tract symptoms. General examination was unremarkable. Digital rectal examination revealed a craggy, firm, ulcerated fixed growth in the rectum, 4 cm from the anal verge between 8 and 4 o'clock (anterior wall of rectum). The prostate was clinically involved, thus suggesting T4 disease. A biopsy of the rectal mass was performed which revealed a moderately differentiated rectal adenocarcinoma. The carcino-embryonic antigen serum marker was within normal limits. A staging CT scan showed a mass involving the anterior and both lateral walls of rectum, with a suggestion of prostatic involvement. A single left-sided peri-rectal node was deemed >1 cm (T4N1M0). A radionucleotide bone scan was negative.

A multidisciplinary approach was performed with chemo-radiotherapy as a first line treatment. The regimen consisted of leucovorin 40 mg intravenous injection each day for 5 days, followed by 5-fluorouracil 600 mg. This was immediately followed by external beam radiotherapy with two parallel opposed fields covering perirectal and pelvic lymph nodes. He received a second course of the same chemotherapy regimen after the radiotherapy. A repeat CT scan revealed a partial response and thus he was offered salvage surgery.

The perioperative period was unremarkable. The estimated operating time was 5.5 h with a blood loss of 1200 ml and a blood transfusion requirement of 3 units. The patient recovered to self sufficient activity and was discharged on the 11^th^ post-operative day.

### Case 2

A woman aged 47 years who in 1998 was found to have a carcinoma of cervix, stage II b treated with radical external beam radiotherapy and brachytherapy presented with pelvic pain and spotting over a period of 2 months. A biopsy of the cervix was performed and it showed squamous cell carcinoma of cervix so chemotherapy with cisplatin single agent was given on a three times a week basis. MRI was performed for staging that showed a disease extending to the lower third of the vagina, and disease was present to the pelvic side wall on the right. A radionucleotide bone scan was negative. A multidisciplinary approach was performed with chemo-radiotherapy as the first line treatment. The regimen consisted of cispaltinum 75 mg intravenous injection each day for 5 days, followed by 5-fluorouracil 200 mg. After that she was offered salvage surgery. The perioperative period was unremarkable. The estimated operating time was 9 h with a blood loss of 1000 ml and any blood transfusion was required. The patient was discharged on the 23^th^ post-operative day.

### Surgical approach

**Figure 1:**
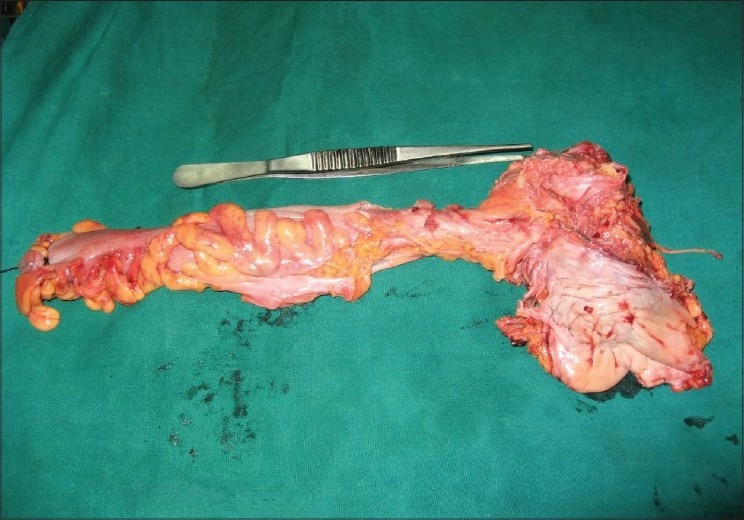
Bladder, prostate and rectum

**Figure 2:**
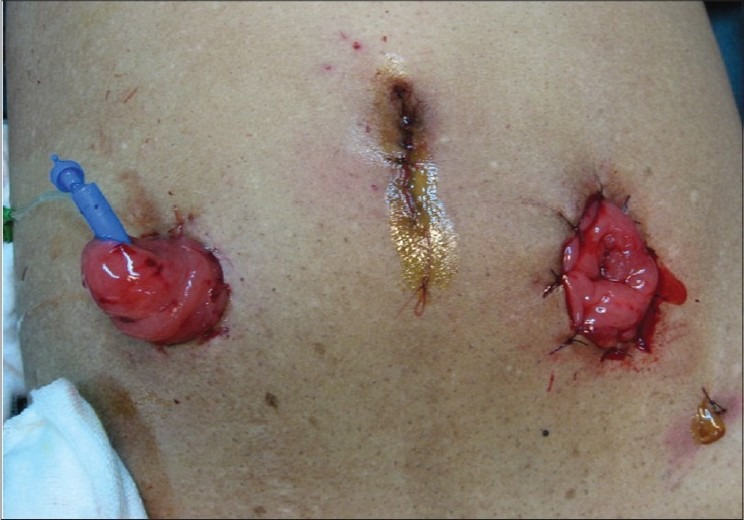
The ileal urostomy and faecal diversion by an end colostomy

Preoperatively (24_h), the patient's bowel was prepared with a purgative and liquid diet. At general anaesthetic induction, broad spectrum antibiotics were administered before placing the subject in a 40 degree trendelenburg position. A five-port transperitoneal laparoscopic total pelvic exenteration was performed, with a perineal excision of the anal margin. The operation consisted of an excision of the sigma-rectum, bladder and prostate in the man and uterus, ovaries in the woman as a single mass [Figure 1], extracted via the perineal wound, followed by an extracorporeal reconstruction for urinary diversion via an ileal urostomy and faecal diversion by an end colostomy [Figure 2]. The pelvis was irrigated with an aqueous tumourcidal betadine solution.

## DISCUSSION

The aim of this study was to determine the immediate surgical outcome following a salvage laparoscopic total pelvic exenteration in a male and in a woman, post-chemo-radiotherapy.

Salvage total pelvic exenteration is often difficult and could be associated with a significant morbidity and mortality.

In 1948, Brunschwig published the first case series of 22 patients who underwent pelvic exenterations; the perioperative mortality rate was 23%. The indications for this innovative radical approach were advanced cancer in the pelvis associated with pain, fistulas or infection in patients, who did not respond to radiotherapy.[1]

Today, 95% of patients undergoing pelvic exenteration for advanced pelvic malignancy are expected to survive surgery[2], and the 5-year survival rate of pelvic exenteration patients (the majority with radiation therapy in their medical history) for recurrent disease has improved to 20-60%.[3–23]

Applying a laparoscopic approach to this group may have advantages for the patient and the surgeon, i.e. less pain, early recovery and magnified views. Laparoscopic salvage surgery is a novel and demanding concept as most surgeons are limited to the application of this approach for open surgery. We have shown the feasibility of carrying out a post-chemo-radiotherapy total pelvic exenteration in a male and a woman. Although not a common indication, many patients are not being offered this option due to the high risk of morbidity. We believe that this procedure can be performed safely with minimal intraoperative complications and without significantly extending the operative time; we still recognize that a prior history of radiation therapy remains a reason for the high postoperative complication rate. Therefore, this option may be offered as an alternative, but patients must be carefully counselled regarding both the benefits and the drawbacks. We recognize that our study has limitations, including the small number of patients and the retrospective data collection, and we conclude that patients with advance pelvic cancer warrant a further study to ascertain which patients would experience maximal benefit with minimal morbidity from this radical surgery, because the goal of radical surgery is to provide an *en bloc* resection of all involved organs and provide a margin-negative resection that can often only be achieved with pelvic exenteration; however, we encourage the use of laparoscopy because of minimal preoperative blood loss, quick recovery, less pain and shorter hospitalization stay. A number of papers have confirmed the absence of significant adverse effects on survival after laparoscopic diagnosis or surgery in pelvic cancers.[24]

As we have technically shown it to be possible, perhaps laparoscopic approaches should be discussed if the teams in these centres are of advanced laparoscopic surgeons working in multi-skilled groups.
